# Implementation of collaborative care for depression in VA HIV clinics: Translating Initiatives for Depression into Effective Solutions (HITIDES): protocol for a cluster-randomized type 3 hybrid effectiveness-implementation trial

**DOI:** 10.1186/s43058-024-00639-z

**Published:** 2024-09-16

**Authors:** Jacob T. Painter, Jeffrey Pyne, Geoffrey Curran, Rebecca A. Raciborski, Shane Russell, John Fortney, Allen L. Gifford, Michael Ohl, Eva N. Woodward

**Affiliations:** 1 HSR&D Center of Innovation Center for Mental Healthcare and Outcomes Research, HSR&D Center of Innovation Center for Mental Healthcare and Outcomes Research, 2200 Fort Roots Dr, North Little Rock, AR 72114 USA; 2 Evidence, Policy, and Implementation Center, Central Arkansas Veterans Healthcare System, 2200 Fort Roots Dr, North Little Rock, AR 72114 USA; 3 Division of Pharmaceutical Evaluation and Policy, College of Pharmacy, University of Arkansas for Medical Sciences, 4301 West Markham St, Little Rock, AR 72205 USA; 4 Department of Psychiatry, College of Medicine, University of Arkansas for Medical Sciences, 4301 West Markham St, Little Rock, AR 72205 USA; 5 Behavioral Health Quality Enhancement Research Initiative (QUERI), Central Arkansas Veterans Healthcare System, 2200 Fort Roots Dr, North Little Rock, AR 72114 USA; 6 Veterans Rural Health Resource Center – Iowa City Veterans Health Care System, 601 US-6 W, Iowa City, IA 52246 USA; 7 Center for Access & Delivery Research and Evaluation (CADRE), Iowa City Veterans Affairs Health Care System, 601 US-6 W, Iowa City, IA 52246 USA; 8 Department of Internal Medicine, University of Iowa Carver College of Medicine, 375 Newton Rd, Iowa City, IA 52242 USA; 9 Center for Healthcare Organization and Implementation Research, VA Boston Healthcare System, 150 S Huntington Ave, Boston, MA 02130 USA; 10 Department of Medicine, Section of General Internal Medicine, Boston University Chobanian & Adevisian School of Medicine and Boston Medical Center, 72 East Concord St, Boston, MA 02118 USA; 11 School of Public Health, Boston University, 715 Albany St, Boston, MA 02118 USA; 12 Center for Implementation Research, Department of Pharmacy Practice, College of Pharmacy University of Arkansas for Medical Sciences, 4301 West Markham St, Little Rock, AR 72205 USA; 13 Department of Psychiatry and Behavioral Sciences, School of Medicine, University of Washington, 1959 NE Pacific Street, Seattle, WA 98195 USA; 14 VA Health Systems Research, Center of Innovation for Veteran-Centered and Value-Driven Care, VA Puget Sound Health Care System, 1660 S Columbian Way, Seattle, WA 98108 USA; 15 Central Arkansas Veterans Healthcare System, 900 S Shackleford Rd, Little Rock, AR 72211 USA

**Keywords:** Implementation, Depression, HIV, Collaborative care, Economic evaluation, Facilitation, Patient engagement, Consumer engagement, Champion, Cost

## Abstract

**Background:**

Depression is the most diagnosed mental health condition among people living with HIV. Collaborative care is an effective intervention for depression, typically delivered in primary care settings. The HIV Translating Initiatives for Depression into Effective Solutions (HITIDES) clinical intervention involves a depression care team housed off-site that supports depression care delivery by HIV care providers. In a randomized controlled trial, HITIDES significantly improved depression symptoms for veterans living with HIV and delivered cost savings. However, no HIV clinics in the Veterans Health Administration (VHA) have implemented HITIDES; as such, it is unclear what implementation strategies are necessary to launch and sustain this intervention.

**Methods:**

This hybrid type-3 effectiveness-implementation trial examines the implementation and effectiveness of HITIDES in 8 VHA HIV clinics randomly assigned to one of two implementation arms. Each arm uses a different implementation strategy package. Arm 1 includes an intervention operations guide; an on-site clinical champion who, with the help of a peer community of practice, will work with local clinicians and leadership to implement HITIDES at their site; and patient engagement in implementation tools. Arm 2 includes all strategies from Arm 1 with assistance from an external facilitator. The primary implementation outcomes is reach; secondary outcomes include adoption, implementation dose, depressive symptoms, and suicidal ideation. We will conduct a budget impact analysis of the implementation strategy packages. We hypothesize that Arm 2 will be associated with greater reach and adoption and that Arm 1 will be less costly.

**Discussion:**

Preliminary work identified implementation strategies acceptable to veterans living with HIV and HIV care providers; however, the effectiveness and cost of these strategies are unknown. While the depression care team can deliver services consistently with high quality, the ability of the depression care team to engage with HIV care providers at sites is unknown. Findings from this study will be used to inform selection of implementation strategies for a broad rollout to enhance depression and suicide care for people living with HIV.

**Trial registration:**

ClinicalTrials.gov ID: NCT05901272, Registered 10 May 2023, https://clinicaltrials.gov/study/NCT05901272

**Supplementary Information:**

The online version contains supplementary material available at 10.1186/s43058-024-00639-z.

Contributions to the literature

- It is well-recognized that those with HIV infections need greater access to mental healthcare for depression. But it is not known how best to support deployment of evidence-based mental healthcare to them.
- This paper describes an implementation trial to roll out depression care in HIV clinics.
- With the fragmentation of care among so many subspecialties, understanding how to roll out mental health support across specialty medical settings is crucial.
- Results will be relevant to mental health and suicide support not only for those with HIV but across many sectors of care for adults receiving care in specialty medical settings.

## Introduction

Depression is the most common mental health condition in patients diagnosed with HIV, with almost 50% diagnosed with depression [1–4]. Depression can lead to a host of negative outcomes [5–11]. Properly treating HIV and its related conditions, such as depression, is a health equity issue, as HIV infection and treatment are negatively impacted by unjust structural factors such as racial discrimination in criminal sentencing, [12, 13] poverty, [14] neighborhood disrepair and segregation, [15, 16] and discrimination in health care [13, 17, 18]. Therefore, HIV is more prevalent among groups experiencing marginalization, including racially and ethnically minoritized individuals, sexual minority men, and people with low incomes [19–21]. Despite availability of efficacious depression treatments, evidence suggests depression is under-diagnosed and under-treated in routine HIV care, similar to primary care settings [22, 23].

The Veterans Health Administration (VHA) is the largest healthcare provider for people living with HIV in the U.S [24]. VHA is also a world leader in implementing collaborative care approaches for improving depression care in primary care [25]. Collaborative care for depression is a multi-faceted, systematic approach to treatment that involves a care manager (typically a nurse) coordinating care between primary care providers, mental health specialists, and other healthcare professionals to optimize patient outcomes. Currently, this high-quality care for veterans with depression in primary care is provided by primary care-mental health integration [26]. However, primary care for veterans living with HIV treated in VHA rests predominantly in HIV clinics rather than in the general medical clinics targeted by primary care-mental health integration; therefore, veterans living with HIV often do not receive the benefits of this service.

HIV Translating Initiatives for Depression into Effective Solutions (HITIDES) is a collaborative care intervention that adapts the primary care collaborative care model for depression treatment to HIV clinics [27]. The depression care manager assesses depression severity and engages patients in treatment planning, following the stepped care model of mental health treatment such that mild to moderate depression is treated with lower intensity services and more severe depression is treated with higher intensity services. The depression care manager summarizes their interactions with the patient in the electronic medical record and the local HIV clinician decides with the patient which of the care steps to pursue. A clinical pharmacist and psychiatrist augment treatment monitoring.

Collaborative care has extremely robust effectiveness data for reducing depression and its negative impacts, inside and outside VHA [28]. In a randomized controlled trial, the HITIDES intervention significantly improved depression outcomes compared with usual care [29]. Moreover, HITIDES operates at a cost-savings compared to the high standard of care in VHA; every dollar invested in HITIDES results in a net gain for VHA in both patient outcomes and healthcare system resources [30]. Despite these benefits, VHA HIV clinics have not implemented HITIDES in routine care. Thus, it is unclear what implementation strategies will be necessary for HIV clinics to adopt this intervention.

In preliminary work, we identified barriers and enablers to implementing HITIDES and we aligned them to domains of the Health Equity Implementation Framework given HIV disparities by income, sexual identity, and racial and ethnic identity [31–33]. Because there are significant advantages to implementing HITIDES, yet also multilevel barriers preventing its adoption, in this study, we aim to support and assess broad implementation of HITIDES by understanding which strategies might best support on-site HIV clinicians and clinic staff to reach more people living with HIV in VHA and adopt depression care team recommendations. We will conduct this study using an equity lens to minimize disparities in receipt of HITIDES between patient groups of different racial and ethnic identities, sexual identities, and income levels by incorporating an equity implementation science framework, using implementation strategies involving patient engagement and equity-grounded reflections at regular meetings with staff, and describing our primary outcome by demographic group. We also plan to assess cost, or budget impact, of implementation strategies, to provide key information to decision makers for scale out.

## Methods

### Study design

**Fig. 1:**
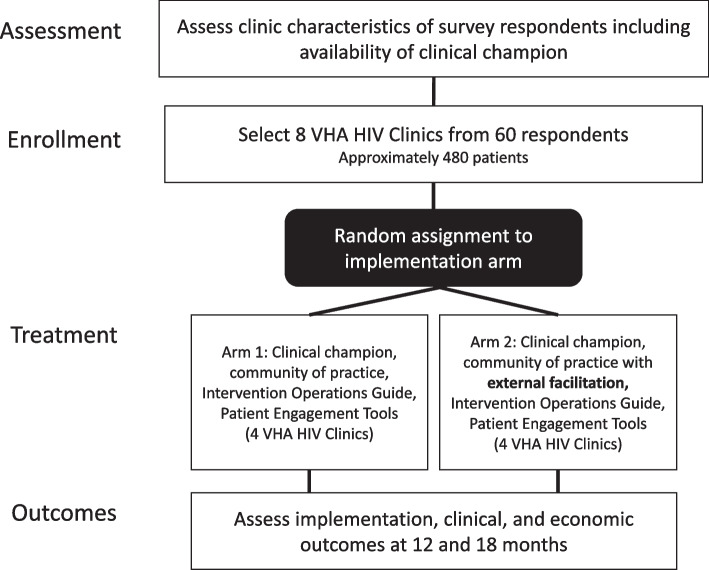
Cluster-randomized trial design

This is a two-arm parallel cluster randomized controlled trial with four VHA HIV clinics in each arm (Fig. 1). We will determine the difference between an implementation strategy package consisting of training a site-level clinical champion, a HITIDES operations guide, patient engagement tools [34], and community of practice [35] (Arm 1) versus that same package with Implementation Facilitation [36], provided by a virtual external facilitator (Arm 2). Our hypothesis is the arm including external facilitation will result in better reach of the HITIDES intervention than the arm without external facilitation because of a higher “dose” of implementation support in Arm 2.

Communities of practice will be hosted separately for sites in Arm 1 and sites in Arm 2 to avoid contamination. Both arms are also supported by educational materials about HITIDES and tools to engage patients in implementation activities [34]. Strategies are described more fully below. We selected outcomes to evaluate public health impact according to the most updated RE-AIM framework [37] including patients reached and their demographic characteristics, site and provider adoption of intervention, patient-level effectiveness, implementation dose including metrics of adherence to strategies and strategy cost, and maintenance of all outcomes at 18-months post implementation. In Aim 1, we will assess our implementation outcomes (reach and adoption) at 12 and 18 months after implementation begins. We will also collect primary data for a summative process evaluation to assess feasibility and acceptability of the implementation strategy packages. In Aim 2, we will assess patient-level effectiveness outcomes (suicidal ideation, depression) at 12 and 18 months after implementation begins using data from the electronic medical record. In Aim 3, we will use primary data collected in Aim 1 and the electronic medical record to evaluate cost of the implementation strategy packages.

### Randomization

The research team will identify sites that (1) have more than 20 Veterans Living with HIV, (2) have adequate PHQ-2 screening data to assess depression prevalence, (3) can identify a clinical champion for implementation activities, (4) are willing to participate, and (5) allow for diversity and balance of clinic characteristics across arms (e.g. rate of referral to specialty mental health for veterans living with HIV and presence of HIV-only specialty clinic versus broad infectious disease clinic). Randomization at the site level has limited ability to completely balance observed and unobserved health-system factors. However, efforts to balance key site characteristics are important; therefore, site-level characteristics thought to impact implementation efforts were identified by building consensus among VHA HIV, Hepatitis, and Related Conditions program leadership and the research team. Data on the following observable site-level characteristics will be collected from these sites during Year 1 of the study: baseline PHQ-2 screen rate, clinic size (unique patients, provider effort, change in enrollment over the past 3 years), and current care manager effort. Based on the five criteria described above and power calculation below, eight eligible sites will be selected. Sites will be grouped into four couplets based on balance of the identified site-level characteristics.

To achieve acceptable power to detect a difference in the primary implementation outcome (reach), a sample of four sites will be needed in Arm 1 and four sites in Arm 2. This assumes an intra-cluster correlation of 0.025, a two-sided Z-test, and an average of 60 participants at each site (*n* = 240 participants per arm). This approach will provide 88% power with alpha = 0.05 to detect a between-group difference in reach of 0.2 (probability of veterans living with HIV with depression who receive HITIDES) between the study arms. Reach is assumed to be 0.2 in Arm 1 and 0.4 in Arm 2. Based on these assumptions, an estimated 144 veterans living with HIV will receive the HITIDES intervention over the course of the study requiring a single depression care team to be provided by clinical operations.

### Implementation strategy selection

Our research team selected implementation strategies based on documented barriers and enablers to implementation, choosing strategies that would hypothetically target change in domains specified by our theoretical determinant framework [32, 33]. The Health Equity Implementation Framework proposes that successful and equitable implementation of evidence-based practices results from facilitation by designated implementation support practitioners (i.e., facilitator, clinical champion) of HITIDES. [38–41] As such, facilitation intensity level was chosen as the implementation strategy to vary between arms, with a higher intensity in one (with external facilitation) and lower intensity in the other (internal clinical champion alone). Implementation support practitioners in both arms must navigate barriers and enablers with patients, providers, and clinic staff in their inner context (HIV clinic) and outer setting (medical facility, VHA healthcare system), all situated within social norms, policies, laws, economic factors, and the built environment of societal context (e.g., HIV stigma, depression stigma, free HIV testing in the U.S., low-cost or no-cost healthcare in VHA per U.S. legislation).

### Implementation strategies

#### Operations guide and patient engagement tools

A HITIDES Operations Guide will include sections on (1) the HITIDES intervention, (2) role of sites and local clinical champions (i.e., a facility-level clinician or nurse involved in HIV care), and (3) implementation tools, including a site self-assessment, decision guide, clinical protocols and algorithms, and provider/patient fact sheets. The clinical champion and facilitator can create other educational materials depending on site needs. Patient engagement tools will be Consumer Voice, an online compendium of modular and nonlinear tools, allowing facilitators and clinical champions to engage in self-guided learning on veteran engagement in implementation activities [34]. The tools are not prescriptive; however, they present core principles and practical ideas for how clinical champions or facilitators might enact the less commonly used “consumer engagement implementation strategies,” such as inviting two veterans living with HIV and depression to inform how patient-facing interaction with HITIDES might work best and to engage in practice “walk-throughs” of HITIDES before official launch. The clinical champion or external facilitator will outreach to VHA patients as veteran consultants who might engage in implementation activities, but not be already served as a recipient of the HITIDES intervention. VHA patients’ engagement and role will vary by site—e.g., some might be existing patients, some may be identified through local community organizations, some may help throughout the process of planning, deploying, and sustaining HITIDES, and others might be involved in only one implementation aspect. The engagement is not focused on the veteran’s own health, but on engaging veterans as consultants or co-leads in HITIDES quality improvement and implementation activities to ensure HITIDES is implemented in a patient-centered way [42]. The HITIDES Operations Guide will be disseminated by VA HIV, Hepatitis, And Related Conditions and provided online at the VHA HIV website for providers, and Consumer Voice tools will be disseminated by the research team.

#### Communities of practice

Communities of practice are groups of clinicians or clinician organizations that work together to implement new practices (i.e., HITIDES) [35]. Key elements of communities of practice are support for members to discuss and engage with one another, sharing knowledge, and enhanced sense of belonging within networks [43]. Aligning existing scholarship on communities of practice as an implementation strategy in this study, communities of practice will be personnel from four sites in a singular arm who meet about enhancing depression care management implementation in HIV clinics (a subject of shared interest), build relationships among members (e.g., clinicians connecting across facilities) to practice integrating new information learned about the intervention [43]. The research team will work with the healthcare system department of VA HIV, Hepatitis, and Related Conditions that informs policy and disseminates best practices to establish monthly community of practice meetings that will operate virtually. Separate communities of practice will be established for each arm to minimize the likelihood of “bleed over” of lessons during Arm 1 into Arm 2, which would unduly impact the performance of (or contaminate) that strategy during Arm 2. Research staff in Arm 1 and the external facilitator in Arm 2 will curate the community of practice. Participation will be monitored by research staff and documented in a log but not controlled.

#### Clinical champion

A clinical champion will be a local physician, nurse practitioner, or physician’s assistant, or nurse in local HIV care who advocates for the HITIDES intervention with local peers. Clinical champions can increase uptake of interventions in health care [44]. A clinical champion can provide ongoing promotion of and education about HITIDES and remind care providers of its presence and value either formally (e.g., presentations at staff meetings) or informally (e.g., individual conversations about HITIDES benefits). Clinical champions can engage middle managers or local leadership to buy in to and support intervention uptake and may work with veterans in quality improvement efforts to garner ideas for implementation and sustainment. Potential champions will be approached after consultation with site HIV care providers and leaders in the Department of VA HIV, Hepatitis, and Related Conditions. Each site, regardless of the arm to which they are assigned, will need a clinical champion.

#### External facilitation

Sites in Arm 2 will receive the implementation strategies from Arm 1 with external facilitation. External facilitation will be provided by a person trained in Implementation Facilitation who is given the HITIDES Operations Guide and Consumer Voice tools for veteran engagement [45]. Facilitation is a process to enable sites to increase uptake of evidence-based practices through building supportive relationships and deploying additional strategies to navigate the implementation process [46]. Although it is difficult to promote sustainable practice change [47–50], facilitation provides an evidence-based approach for achieving such change [51–55]. The external facilitator will interact with sites virtually via telephone and videoconference technology, including facilitating in the community of practice. By working with leadership, clinical champions, and veterans, facilitators can better identify and manage issues in the outer context, such as regional leadership engagement concerns, and issues related to patient-level barriers such as limited literacy or suggestions on care flow for HITIDES. The facilitator will use or support sites to use implementation strategies other than those already named in Arm 1 to promote expansion of HITIDES with an eye toward health equity using ongoing assessment aligned to the Health Equity Implementation Framework, patient engagement tools, and regular equity-grounded reflections at meetings. The facilitator will provide expertise about the implementation process and content subject matter expertise about HITIDES, connecting to other experts as needed [41]. Sustainability Action Plans will be completed in each site participating in the arm with external facilitation since this is a standard recommendation for facilitators. The external facilitator will be funded and monitored by research staff, although this person will not conduct formal research activities.

### Common data sources across aims

There are two data sources across our multiple study aims—see Table 1. They are described here, and then we present data sources specific to each Aim in later sections.

**Table 1:** Secondary data sources used across aims

Construct	Instrument / Data Source	Baseline	12 mos	18 mos
**Baseline data**				
Depression screen	PHQ-2 / VHA Electronic Health Record	✓		
Demographics	VHA Electronic Health Record	✓		
Physical comorbidity	ICD-10 / VHA Electronic Health Record	✓		
Depression history	ICD-10 / VHA Electronic Health Record	✓		
Mental health diagnoses	ICD-10 / VHA Electronic Health Record	✓		
**Implementation outcomes (Aim 1)**				
Reach	Visit codes / VHA Electronic Health Record		✓	✓
Adoption	Provider file, pharmacy file, consult file & stop code / Behavioral Health Laboratory & VHA Electronic Health Record		✓	✓
**Effectiveness outcomes (Aim 2)**				
Depressive symptoms	PHQ-9 / Behavioral Health Laboratory	✓	✓	✓
Suicidal ideation	C-SSRS/ Behavioral Health Laboratory	✓	✓	✓
**Budget impact data (Aim 3)**				
VHA service utilization	Outpatient & pharmacy / MCA	✓	✓	✓
VHA costs	Outpatient & pharmacy / MCA	✓	✓	✓

*C-SSRS* Columbia – Suicide Severity Rating Scale, *ICD* International Classification of Diseases, *MCA* Managerial Cost Accounting, *PHQ* Patient Health Questionnaire, *VHA* Veterans Health Administration

#### VA Corporate Data Warehouse (CDW)

The VA Corporate Data Warehouse (CDW) is a relational database organized into a collection of data domains derived from the VHA electronic health record. It contains records of inpatient and outpatient care, including dates and location of care and ICD-10 codes associated with care. We will use electronically abstracted demographic data, diagnostic codes, pharmacy, and lab data from CDW. These data will be used for the identification and characterization of eligible participants. Site-level aggregation of these data will be used for most implementation outcomes. The potential participant pool will include those with an appointment in a VA HIV or infectious disease clinic and a positive screen on Patient Health Questionnaire-2 (PHQ-2) in the past 18 months.

#### Behavioral Health Lab

The Behavioral Health Laboratory is a VHA clinical service that assesses and summarizes mental health symptoms [56] and operates through a platform clinicians use during care. This platform allows a depression care manager to assess patient symptoms over time (i.e., measurement-based care), assists in making evidence-based treatment recommendations and allows for these recommendations to be communicated back to the patient care team. The Behavioral Health Laboratory is currently used in VHA primary care collaborative care for depression; thus, it is well suited for use in the HITIDES intervention.

### Specific aim 1 procedures, data collection, and analysis

#### Procedures

The HITIDES intervention will be delivered by the depression care team who work and are supervised in routine clinical operations. As patients screen positive for depression before or during medical appointments in HIV clinics, the depression care manager will coordinate with the HIV clinician on whether to offer care management to those veterans or if medication consultation is needed from the clinical pharmacist and/or psychiatrist. Throughout the implementation period, the research team will collect data to document the implementation process. To assess recruitment capability of the patient engagement tools, Consumer Voice, to engage and maintain patient consultants in the implementation process, research staff will conduct a brief phone call interview with those that declined or dropped out to understand why. At the end of the 12-month period, the research team will compare implementation strategy packages by assessing reach and adoption of HITIDES between arms. Also at this time, the research team will collect data for the summative process evaluation of implementation in both arms to interpret findings related to reach, adoption, and implementation dose (described below).

#### Reach

The primary study outcome and measure of reach for this study is proportion of eligible patients receiving the HITIDES intervention. The denominator of the measure (eligible patients) will a site-level estimate for patients with positive depression screens in each clinic on the PHQ-2. The numerator (patients reached by HITIDES intervention) will be the number of eligible patients at each site who receive HITIDES. Receipt of HITIDES will be defined as documentation of an initial consultation with the depression care manager plus at least one follow-up call. We will also document Reach Equity—differences between sociodemographic groups of those screening positive for depression who were not reached by HITIDES and those who screened positive and were reached. Significant differences between demographic groups might suggest reach inequities and we can report the effect size, thus, analyzing reach through an equity lens. Among veterans reached by HITIDES, we will assess demographic characteristics using administrative data available, including racial identity, ethnicity, age, annual income, sexual identity, gender identity, and rurality of residence [37]. Reach will be calculated as a proportion at 12 and 18 months. The primary comparison of interest, and the one on which the study is powered, is the comparison of reach at 12 months in Arm 1 vs. Arm 2. We will analyze demographic characteristics of veterans reached by HITIDES using administrative data available to calculate percentages, means, and standard deviations where relevant.

One additional analysis will estimate the impact of implementation dose, our hypothesized mechanism of action, on reach. We will assess implementation dose as time spent on implementation by clinical champions, community of practice coordinators, and in Arm 2, the facilitator.

#### Adoption

Adoption of the HITIDES intervention will provide an estimate of provider *and* site engagement with HITIDES. The denominator for *provider*-level adoption will be the historical PHQ-2 positive rate of each provider’s current panel of veterans living with HIV. The denominator of the *site*-level adoption measure (eligible patients) will be a site-level estimate for patients with positive depression screens in each clinic. The numerator for adoption at the *site and provider* level will be number of referrals to the depression care team from local HIV care providers or clinic staff and from each individual provider within a given HIV clinic. Adoption will be assessed at 12 and 18 months as a proportion.

Adoption will be further evaluated by the extent to which HIV clinicians use recommendations made by the depression care management team. This measure will be aggregated for individual providers and at the site level. Data for this measure will be gathered from Behavioral Health Laboratory, where the depression care manager will record psychotherapy and pharmacotherapy recommendations made by the depression care team. Each of these recommendations will be categorized by stop code (in the case of psychotherapy) and drug name, strength, pill count, and days’ supply for pharmacotherapeutic recommendations. Once all recommendations have been classified, they will be compared to the corresponding fields in the electronic health record to determine if recommendations were used by the HIV clinician.

#### Implementation dose

##### Survey: pragmatic implementation strategy reporting tool

This tool will be administered in the form of an electronic survey to the external facilitator and clinical champions at each site in both arms to report implementation strategies used throughout implementation [57]. This will be administered up to three times throughout the study. We will calculate frequencies of each implementation strategy used across sites, and document reasons why. We will add one item asking clinical champions their estimated time spent weekly on implementation efforts. We will create a median or average score to describe number of implementation strategies across clinical champion sites in each arm.

##### Implementation facilitation time-tracking log

The external facilitator will complete this time-tracking log in Arm 2, which prompts them to identify activities, personnel, sites, and time spent engaging in facilitation. This tool was developed by VHA Behavioral Health Quality Enhancement Research Initiative and has been successfully used to estimate time and cost of facilitation [58]. Data collected from the time-tracking log in Aim 1 will be used to identify time and activities spent during facilitation. These data will also be analyzed in Aim 3 to estimate cost of facilitation.

##### Community of practice attendance list and topics

We will track attendance of people at each community of practice and their topics and dates across arms. We will summarize counts of people in attendance in community of practice sessions over time and by site and by study arm at community of practices as a descriptive variable of this implementation dose. These data will also be analyzed in Aim 3 to estimate cost of facilitation.

#### Summative process evaluation of patient engagement in implementation tools (Consumer Voice)

The following measures and analyses are focused on acceptability and feasibility of Consumer Voice tools for patient engagement in implementation (not the HITIDES intervention). We hypothesize Consumer Voice will be acceptable and feasible to implementation support practitioners across Arms. The goal of Consumer Voice is not aimed at increasing patients’ activation in their own healthcare, but to help implementation support practitioners at each site operationalize implementation strategies already planned in both arms in a way that is patient-centered or determine new implementation strategies from patient input.

##### Survey: acceptability and feasibility measures

We will administer a 12-item survey consisting of three subscales, each with four items each about Consumer Voice tools for patient engagement (not the HITIDES intervention): the Acceptability of Intervention Measure, Intervention Appropriateness Measure, and Feasibility of Intervention Measure. These are psychometrically valid and reliable [59]. This will be administered as an electronic survey up to three times throughout the study to clinical champions and the external facilitator. We will sum scores on this questionnaire for each person, and aggregate them to produce a median, average, and range across sites.

##### Interview with clinical champions and facilitator

During this interview at the end of implementation, we will discuss 1) acceptability, feasibility of Consumer Voice and 2) any elaboration to understand all elements of additional strategies deployed reported on the Pragmatic Implementation Strategy Reporting Tool. Interviews will be conducted according to a brief, semi-structured guide, conducted via telephone or video conference, and audio recorded. Two research staff will conduct qualitative analysis of these audio recorded data at the 12-month period (end of active implementation). Interview notes will be prioritized for analysis, but data may be transcribed if needed. Specifically, we will use template analysis based on questions from the interview guide. Ideas repeated by two or more participants will be reported and synthesized with other related repeating ideas into themes.

During these interviews, we will also ask how many VHA patients were approached and how many engaged as patient consultants to the implementation process. First, we will calculate percentage of veterans who engaged at all out of all veterans approached, and then we will calculate percentage of veterans who remained engaged throughout the implementation phases out of all veterans who started engagement.

##### Interview guide: patient experience in implementation activities

We will contact VHA patients who were invited to engage as patient consultants in implementation using Consumer Voice. The purpose is to document experience and burden of engagement. We will collect demographic information that may be associated with the decision to participate. For those who chose to engage and those who engaged but dropped out, we will collect information soon after they stop engaging about costs incurred to engage (e.g., travel expenses, internet for virtual meetings). We will also ask open-ended questions about their experience being engaged in implementation activities, focusing on acceptability. Those who declined to participate or dropped out, they will be asked an open-ended question about their reasons for choosing not to participate. For the qualitative data generated by this interview, two research staff will conduct qualitative analysis of these audio recorded data. Interview notes will be prioritized for analysis, but data may be transcribed if needed. Specifically, we will use template analysis, creating our template based on questions from the interview guide. Ideas repeated by two or more participants will be reported and synthesized with other related repeating ideas into themes. We will compute descriptive statistics for the data generated from demographic questions.

##### Sustainability action plan review

We will review Sustainability Action Plans completed in each site by clinical champions (Arm 1) and facilitators (Arm 2). We will compare completed Sustainability Action Plans across sites for integration of patients in planned sustainment activities. We will code for three criteria within each Sustainability Action Plan: 1) planned communication between patients and sites, 2) patients involved in developing or reviewing the plan, and 3) patients being sampled for a metric of sustainability (e.g., patient satisfaction, data review of patient receipt of depression care). These criteria were informed by a subscale of consumer engagement in a reliable, quantitative sustainability measure [60]. Two research staff will code Sustainability Action Plans using a predetermined rubric and assign each plan a total score.

### Specific aim 2 procedures, data collection, and analysis

#### Effectiveness

Because effectiveness of HITIDES was documented in a previous randomized controlled trial, effectiveness is not the primary outcome of this study. However, questions remain regarding the robustness of the effect of the intervention on depression across sites receiving different implementation strategy packages. Additionally, given the concern for suicide in the veteran population and the relationship between suicide and depression, an examination of the effects of HITIDES on suicidal ideation is warranted. We hypothesize that Arm 2 sites receiving virtual external facilitator will improve fidelity, and thus HITIDES recipients will experience improved depressive symptoms and reduced suicidal ideation.

##### Depression

The Patient Health Questionnaire (PHQ) is used in research and screening for various mental health disorders [61]. The PHQ-9 has high sensitivity and specificity for major depression diagnoses with higher scores indicating greater symptom severity. The depression care manager will conduct the PHQ-9 at baseline; (PHQ-9 ≥ 10 required to continue intervention) and at each follow-up call for HITIDES patients. Change in depression severity will be examined as a continuous variable comparing baseline PHQ-9 scores with PHQ-9 scores at 12 and 18 months. We define depression response as a 50% reduction from baseline PHQ-9 score and depression remission as a PHQ-9 score at 12 or 18 months < 5.

##### Suicidal ideation

The Columbia-Suicide Severity Rating Scale (C-SSRS) is a valid, reliable measure of suicidal ideation, intent, and risk sensitive and specific to identifying suicidal ideation and behavior and change over time with higher scores signaling more severe suicide risk [62]. The current VHA process for suicide risk assessment is for providers to administer the PHQ-2 plus the PHQ-9 suicidal ideation question, which, if positive, prompts the administration of C-SSRS. We will code patients as not having suicidal ideation if the C-SSRS score equals 0. At baseline and each follow up call, if veterans endorse suicidal thoughts, the depression care manager will administer the C-SSRS consistent with routine practice. We will consider suicidal ideation reduction as a decrease of one or more in the C-SSRS score and suicidal ideation remission as a move from any nonzero score to zero.

Generalized linear mixed models will account for clustering patients and providers within sites. The site will be specified as a random effect, and the period will be defined as a fixed effect. The effectiveness regression will be estimated using the patient as the unit of analysis. Case mix control variables will include baseline depression severity, psychiatric comorbidity, period of wartime service, and service-connected disability.

### Specific aim 3 procedures, data collection, and analysis

#### Implementation dose

As one component of implementation dose according to RE-AIM [37], we will estimate the budget impact of HITIDES implementation strategies –clinical champions, community of practice, patient engagement tools (Consumer Voice) and external facilitation [63]. Economic analyses provide crucial information to policymakers who seek to understand whether implementation is feasible from a cost perspective and what potential modifications might make it so [64]. To accomplish Aim 3, we will follow steps developed by the International Society for Pharmacoeconomics and Outcomes Research. A budget impact analysis describes the “current” environment before HITIDES implementation strategies are introduced and “new” environment after introduction of the strategies [65]. Data for these estimations will be extracted primarily from Managerial Cost Accounting (MCA) database. Primary data collection from Aim 1 will be used to estimate implementation costs. Thus, the evaluation team will collect these data from implementation support practitioners in both arms—external facilitator and clinical champions. Once desired data on the current and new environments are collected and compiled, we will conduct an analysis estimating the budget impact of HITIDES implementation strategy packages at participating sites [66]. Information on the current pre-implementation environment will be analyzed from the payer's (VHA) perspective.

The VHA-designated MCA System is a derived database built from standard VHA data sources, including the CDW [67]. MCA data allow for comparison of cost and healthcare utilization characteristics. Healthcare costs during the trial will be assessed using MCA, which is based on the activity-based cost allocation method and includes fixed direct, variable direct, and fixed indirect costs. Resources and utilization will be estimated to describe the current price of care in the pre-implementation environment. Outpatient and pharmacy utilization and cost data for eligible patients (screened positive on depression via PHQ-2) at participating sites will be collected using CDW data. Outpatient utilization and cost data will be taken from the MCA outpatient file and summed at the site level.

For the primary analysis, encounters will be organized in the following groups by primary stop code field: primary care, mental health, infectious disease, other specialty care, ancillary, and others. Outpatient VHA medication costs will be assessed using MCA pharmacy data; prescription drug use will be divided into HIV-related, mental health-related, and other. To estimate the cost of implementation, we will use MCA data and time devoted to implementation activities from clinical champion and facilitator time-tracking. Based on these data, appropriate salary and fringe rates can be applied to each activity to estimate the cost of implementing and delivering the intervention. The base-case analysis estimates the difference in cost of illness before and after HITIDES implementation plus the cost of implementation under each condition.

## Discussion

In this hybrid type 3 effectiveness-implementation study, we aim to understand how varying intensities and doses of implementation strategies impact the implementation success of the HITIDES intervention in HIV/infectious disease clinics and subsequent clinical outcomes. A key area of exploration will be the extent to which site clinical champions can effectively use tools for patient engagement in implementation activities. Additionally, evaluating the financial implications of these implementation strategies will provide valuable insights for decision-makers contemplating adoption of collaborative care for depression models in specialty medical clinics, especially for clinics serving people living with HIV. We do not anticipate direct harm from this research however, mechanisms are in place to monitor, report, and address issues that may arise.

Methods to balance rigor and practicality have been critical in the design of this investigation. A key scientific challenge addressed was ensuring the independence of facilitators from evaluators to avoid potential biases. To this end, distinct teams for evaluation and implementation facilitation have been established, along with a project coordinator serving as an intermediary. This coordinator's role is to facilitate essential communications that are pertinent to the functioning of both teams, excluding discussions about the trial's progress or preliminary findings. This structure is consistent with successful approaches observed in previous hybrid type 2 or 3 effectiveness-implementation studies [68]. Additionally, aligning financial and human resources with clinical partners for this hybrid type 3 study has been critical to ensure that personnel delivering HITIDES are integrated within existing clinical services, not the research team. This approach, facilitated through collaboration with potential sites and funding agencies, increases likelihood of long-term sustainment of the depression care team after the study is completed, illustrating a commitment by some clinics to overcome the barriers associated with staffing such initiatives.

## Supplementary Information

Supplementary Material 1.

Supplementary Material 2.

## Data Availability

The datasets used and/or analyzed during the current study will be available from the corresponding author on reasonable request.

## Abbreviations

CDW: Corporate Data Warehouse
C-SSRS: Columbia-Suicide Severity Rating Scale
HITIDES: HIV Translating Initiatives for Depression into Effective Solutions
HIV: Human Immunodeficiency Virus
HSR&D: Health Systems Research & Development
MCA: Managerial Cost Accounting
ME: Marginal Effects
VHA: Veterans Health Administration
